# Kosmetische und funktionelle Ergebnisse nach Resektion kutaner Neurofibrome bei Neurofibromatose I: Die Operation von Neurofibromen führt zu einer Verbesserung der Lebensqualität bei moderaten Komplikationen

**DOI:** 10.1007/s00105-023-05220-8

**Published:** 2023-09-20

**Authors:** Benjamin Walz, Vanessa Pfefferle, Hans-Martin Häfner, Lukas Kofler

**Affiliations:** 1 Universitätshautklinik Tübingen, Liebermeisterstr. 25, 72076 Tübingen, Deutschland; 2 Zentrum für Seltene Hauterkrankungen, Liebermeisterstr. 25, 72076 Tübingen, Deutschland; 3 Hautzentrum skin+more, Holzmarkt 6, 88400 Biberach an der Riss, Deutschland

**Keywords:** Neurofibromatose, Dermatochirurgie, Lokalanästhesie, Komplikationen, Lebensqualität, Neurofibromatosis, Dermatosurgery, Local anesthesia, Complications, Quality of life

## Abstract

**Hintergrund:**

Neurofibromatose Typ 1, auch Morbus Recklinghausen genannt, ist ein neurokutanes Tumorsyndrom, welches genetisch bedingt ist und mit teils ausgeprägtem Befall des Integuments mit Neurofibromen (Nervenscheidentumoren) einhergeht. Kutane Neurofibrome können für Patienten sehr belastend sein und tragen v. a. an äußerlich sichtbaren oder funktionell relevanten Körperstellen zu einer Verminderung der Lebensqualität bei. Die vorliegende Arbeit soll zeigen, inwiefern durch eine Resektion kutaner Neurofibrome eine Verbesserung der Lebensqualität bewirkt werden kann.

**Methodik:**

Wir führten eine retrospektive Datenerhebung per Fragebogen zur Lebensqualität vor und nach der Operation kutaner Neurofibrome in der Universitätshautklinik durch. Verwendet wurden ein adaptierter dermatologischer Lebensqualitätsindex sowie ein in unserer Hautklinik eingesetzter postoperativer Fragebogen zu Komplikationen und Patientenzufriedenheit. Zudem wurden Daten der Patienten aus Arztbriefen, Operationsberichten und Ambulanzdokumentationen entnommen. Befragt wurden 30 Patienten mit Neurofibromatose Typ 1, die innerhalb der Jahre 2016 bis 2020 in der Hautklinik Tübingen ambulant oder stationär operiert wurden. Die Befragungsergebnisse wurden statistisch ausgewertet und als absolute und relative Häufigkeiten dargestellt.

**Ergebnisse:**

Unsere Studie zeigt eine Verbesserung der Lebensqualität nach operativer Therapie kutaner Neurofibrome, hinsichtlich Einschränkungen im Alltag, Befangenheit, Einschränkungen der Kleiderwahl und der Freizeitgestaltung. Dabei kam es bei der Mehrheit der Patienten zu keinem neuen Auftreten von Neurofibromen im Operationsgebiet; Komplikationen wie Nachblutungen oder Wundinfekte fallen in unserer Studie moderat bis gering aus.

**Diskussion:**

In Relation zu hoher Zufriedenheit mit dem operativen und kosmetischen Ergebnis sowie positivem Einfluss auf die Lebensqualität spricht dies für ein günstiges Risiko-Nutzen-Verhältnis der Resektion kutaner Neurofibrome bei Neurofibromatose I.

**Zusatzmaterial online:**

Zusätzliche Informationen sind in der Online-Version dieses Artikels (10.1007/s00105-023-05220-8) enthalten.

## Abstract (en)

**Background:**

Neurofibromatosis type 1, also known as Recklinghausen’s disease, is a neurocutaneous tumor syndrome that is genetically determined and is associated with infestation of the integument with neurofibromas (nerve sheath tumors). The occurrence of neurofibromas can be very stressful for patients and often contributes to a reduced quality of life for patients, especially if externally visible body parts are affected. The aim of this study is to show to what extent the resection of cutaneous neurofibromas can improve patients’ quality of life.

**Materials and methods:**

For this study, we conducted a retrospective data collection via questionnaire on the quality of life before and after the surgical removal of cutaneous neurofibromas at the Department of Dermatology, University Hospital of Tübingen. An adapted dermatological quality of life index and a postoperative questionnaire on patient satisfaction were used. In addition, patient data were taken from doctor’s notes, surgical reports, and outpatient documentation. A total of 30 patients with neurofibromatosis type 1 who underwent inpatient or outpatient surgery for cutaneous neurofibromas at the Tübingen dermatology hospital between 2016 and 2020 were surveyed. The survey results were statistically analyzed and represented as absolute and relative frequencies.

**Results:**

Our study indicates an improved quality of life after surgery for cutaneous neurofibromas, especially regarding limitations in everyday life, self-consciousness, the choice of clothing, and leisure activities. The majority of our patients showed no new occurrence of neurofibromas in the surgical area and postoperative bleeding or wound infections were rare.

**Conclusion:**

In relation to the high level of satisfaction with the surgical and cosmetic results and also the positive influence on quality of life, our study indicates a favorable risk–benefit ratio for the resection of cutaneous neurofibromas in neurofibromatosis I.

Die Neurofibromatose Typ 1 stellt als neurokutanes Tumorsyndrom v. a. bei ausgedehntem Befall des Integuments mit multiplen Nervenscheidentumoren (Neurofibromen) eine große Belastung für die betroffenen Patienten dar. Bis dato existiert keine ursächliche Heilungsmöglichkeit der Neurofibromatose Typ 1, daher bleibt die operative Exzision der kutanen Neurofibrome die empfohlene Therapie. Im Fokus bei der Operation stehen auch kosmetische und funktionelle Aspekte, um eine Verbesserung der Lebensqualität zu erzielen.

Die Neurofibromatose Typ 1 (NF1), auch Morbus Recklinghausen genannt, ist ein neurokutanes Tumorsyndrom. Die Erkrankung ist genetisch bedingt, folgt einem autosomal-dominanten Erbgang und kann sich an multiplen Organen manifestieren. So kann beispielsweise die Iris, das zentrale Nervensystem oder Knochen betroffen sein [1].

Die diagnostischen Kriterien der NF1 umfassen (mindestens 2 müssen für die Diagnosestellung zutreffen; [1]):

- mindestens 6 Café-au-lait-Flecken (mehr als 5 mm Durchmesser präpubertär, mindestens 15 mm postpubertär),
- axilläres oder inguinales Freckling (sommersprossenartige Flecken),
- Optikusgliom,
- mindestens 2 Lisch-Knötchen (Irishamartome),
- mindestens 2 Neurofibrome oder ein plexiformes Neurofibrom,
- Knochenveränderungen (Keilbeinflügel, lange tibiale Pseudoarthrose),
- 1 erstgradiger Verwandter mit NF1.

Die Inzidenz der NF1 liegt bei knapp 1:3000 Geburten [1, 2], und die Prävalenz beträgt 1:4560 [3].

Namensgebendes Kennzeichen ist das Auftreten von benignen Nervenscheidentumoren, den Neurofibromen [1]. Diese können an ausgedehnten Hautarealen in großer Stückzahl auftreten und somit für betroffene Patienten sehr stigmatisierend sein. Dies trägt v. a. an äußerlich sichtbaren oder funktionell relevanten Körperstellen zu einer Verminderung der Lebensqualität bei [2–4]. Ebenso ist ein negativer Einfluss der NF1 auf Bildung, Arbeit und Einkommen beschrieben worden [5].

Da nach wie vor keine ursächliche Behandlung der NF1 zur Verfügung steht, ist die operative Therapie der kutanen Neurofibrome weiterhin Therapie der Wahl. Hier stehen als Möglichkeiten die Exzision, Horizontalexzision, Kürettage oder Dermabrasion zur Verfügung. Bei kleineren Neurofibromen oder unterstützend zur operativen Therapie bei größeren Tumoren kommt eine Ablation mittels CO_2_-Laser infrage [6, 7]. Größere oder schwer zugängliche betroffene Areale oder Wammen-artige Neurofibrome können eine technische Herausforderung darstellen und ggf. ein mehrzeitiges Vorgehen erfordern [7]. Bei der operativen Therapie spielen ästhetische, aber auch funktionelle Gesichtspunkte eine Rolle.

Während die Beeinträchtigung der Lebensqualität durch NF1 in der Literatur gut etabliert ist [8, 9] und sich auch Studien zu Rezidivraten nach Operation finden [10, 11], ist der Einfluss der Operation auf die Lebensqualität noch wenig erforscht [12]. Ziel dieser Studie war daher, Fragebogen-basiert retrospektiv zu erfassen, welche Auswirkungen die Operation kutaner Neurofibrome auf die Lebensqualität der Patienten mit einer NF1 hat und welche Komplikationen dabei auftreten.

## Methoden

### Patientenkollektiv

Für die Studie wurden Patienten, die innerhalb der Jahre 2016 bis 2020 in der Hautklinik Tübingen bei kutanen Fibromen einer NF1 ambulant oder stationär operativ behandelt wurden, eingeschlossen. Wir führten die Datenerhebung retrospektiv per Fragebogen zur Lebensqualität durch. Nach Rücksendung der durch die Patienten beantworteten Fragebögen sowie erfolgtem schriftlichem Einverständnis wurden die Bögen anonymisiert ausgewertet. Insgesamt kamen 45 Patienten für unsere Studie infrage und wurden angeschrieben, bei einer Responderquote von 66,6 % konnten 30 Patienten in die Studie eingeschlossen werden.

Die Datenerhebung und Auswertung erfolgte mit Zustimmung der zuständigen Ethikkommission sowie unter Berücksichtigung der Deklaration von Helsinki (1975). Außerdem wurden relevante Zusatzinformationen aus der Patientenanamnese miterfasst (Histologie, Befunde aus der Humangenetik, Operationsberichte und Folgeeingriffe); hierfür wurden Arztbriefe und Ambulanzdokumentationen aus unserer Klinik herangezogen. Die Erhebung und Auswertung der Daten aus den Fragebögen erfolgten durch Dr. Benjamin Walz. Auch wurden patientenspezifische Parameter wie Alter und Geschlecht erhoben. Diese patientenbezogenen Daten wurden durch Dr. Vanessa Pfefferle und Dr. Benjamin Walz erhoben.

Alle in unserer Klinik im Untersuchungszeitraum durchgeführten Eingriffe erfolgten in Tumeszenzlokalanästhesie (Lösung aus Lidocain, Ropivacain sowie Epinephrin, zu einer Gesamtkonzentration von 0,05, 0,11 oder 0,21 % in Jonosteril [13]). In der Hautklinik Tübingen erfolgt ein multimodaler operativer Ansatz zur Entfernung der Neurofibrome, bei dem je nach Größe, Lokalisation und Verteilung der Neurofibrome verschiedene Operationstechniken zum Einsatz kommen. So kann eine komplette Exzision der Neurofibrome mit anschließendem Wundverschluss durch Dehnungs- oder Verschiebelappenplastiken, bei kleineren Defekten auch durch eine primäre Wundnaht durchgeführt werden. Bei spannungsreichen oder behaarten Lokalisationen wie distalen Extremitäten oder der Kopfhaut kamen extrakutane, sonst resorbierbare intrakutane Butterflynähte zum Einsatz [14]. In seltenen Fällen erfolgten auch Adaptationsnähte (partieller Wundverschluss unter Spannung) oder eine sekundäre Wundheilung. Pro Sitzung erfolgten typischerweise mehrere Exzisionen.

Neben einer Komplettexzision der Neurofibrome wurden – v. a. bei großflächigen Arealen mit multiplen kleineren Fibromen – auch die Abtragung mittels Scherenschlag (tangentiale Abtragung der Epidermis, teils mit anschließender Entfernung des dermalen Anteils des Fibroms mit der Pinzette) oder Stanzbiopsien mit und ohne Naht durchgeführt.

### Fragebogen

Die Fragebögen bestanden aus verschiedenen Abschnitten, welche in leicht abgeänderter Form zur Erfassung der Lebensqualität bei Hauterkrankungen verwendet werden. Dazu zählt in erste Linie ein modifizierter DLQI(Dermatologic Life Quality Index)-Fragebogen. Der genaue Aufbau des abgewandelten DLQI ist Tab. 2 sowie dem Anhang unter Punkt 1.1 zu entnehmen. Darüber hinaus gibt es einen in unserem Hause eingesetzten postoperativen Fragebogen zur Erhebung postoperativer Ergebnisse, Komplikationen und Patientenzufriedenheit (Anhang 1.2). Die Patientenzufriedenheit wurde als Multi-Item-Fragebogen mit Fragen zur Zufriedenheit mit dem Operationsergebnis insgesamt und dem ästhetischen Ergebnis, jeweils auf einer nominalen Skala (sehr zufrieden, zufrieden, ausreichend zufrieden, nicht zufrieden, sehr unzufrieden) sowie der Weiterempfehlung einer Operation in unserem Hause (ja/nein), abgefragt. Auch wurde in diesem Fragebogen nach Komplikationen wie postoperative Schmerzen, Nachblutungen oder Wiederauftreten von Neurofibromen im Operationsgebiet gefragt.

### Statistische Aufarbeitung

Die statistische Auswertung erfolgte über Microsoft Excel® sowie JMP® von SAS. Hier wurden die Ergebnisse der Fragen als nominale Parameter (ja/nein bzw. stark/gering etc.) digital aufgelistet und alle Nennungen eines Parameters pro Frage summiert. Diese Summe wurde stets durch die Gesamtzahl aller 30 Probanden dividiert (auch bei fehlenden Werten bei Nichtbeantwortung einzelner Fragen wurde durch 30 dividiert), um den Prozentanteil des jeweiligen Parameters pro Frage zu erhalten. Die Darstellung der Ergebnisse erfolgt prozentual und auf eine Stelle nach dem Komma gerundet.

## Ergebnisse

### Patientenkollektiv

Angaben zum Patientenkollektiv sind in Tab. 1 aufgeführt.

**Table 1:** 

**Patienten**	**Anzahl**	**Durchschnittsalter in Jahren**		**Ambulant**	**Stationär**	**Ambulant und stationär**	
Männlich	13	35,7		11	1	1	
Weiblich	17	43,2		9	5	3	
–	**0 bis 6 Monate**	**6 bis 12 Monate**		**1 bis 2 Jahre**	**2 bis 5 Jahre**	**>** **5 Jahre**	
Erstmanifestation Neurofibrome bei NF1 vor Operation	0 %	0 %		0 %	6,7 %	93,3 %	
Erstvorstellung wegen Neurofibromen bei NF1 vor Operation	10 %	6,7 %		3,3 %	3,33 %	76,7 %	
–	**Rumpf**	**Arme**	**Beine**	**Gesicht**	**Hals**	**Sonstige Lokalisation am Kopf**	**Andere**
Lokalisation der Neurofibrome	90 %	66,7 %	60 %	60 %	50 %	43,3 %	3,3 %

*NF1* Neurofibromatose Typ 1

Unter den Teilnehmern waren 17 Personen weiblich und 13 Personen männlich. Das Durchschnittsalter zum Zeitpunkt der ersten Operation im genannten Zeitraum betrug insgesamt 39,9 Jahre, wobei die männlichen Patienten mit 35,7 Jahren etwas jünger waren als die weiblichen Patienten mit 43,2 Jahren. Zwei Teilnehmer waren zum Zeitpunkt ihrer Operation in unserem Hause minderjährig, hier erfolgten Zustimmung und Beantwortung der Fragebögen durch die Eltern.

Elf Patienten wurden im angegebenen Zeitraum einmalig und 19 Patienten mehrmals operiert. Bei den meisten Sitzungen erfolgte die Entfernung mehrerer Neurofibrome. Ein Patient wurde sogar 20-mal bei insgesamt 470 Läsionen im Untersuchungszeitraum operiert. Allerdings lässt sich gerade bei großflächigerem Befall, wobei hier vorwiegend Methoden, wie z. B die Kürettage an größeren Arealen, zum Einsatz kamen, teils keine genaue Anzahl der entfernten Neurofibrome angeben. Eine genaue Übersicht ist im Anhang Punkt 2 tabellarisch aufgeführt.

Bei 28 unserer 30 befragten Patienten trat die Neurofibromatose an der Haut in Form kutaner Neurofibrome mehr als 5 Jahre vor der ersten Operation in unserem Hause auf (93,3 %). Bei 2 der Patienten (6,7 %) waren es zwischen 2 und 5 Jahren. Die ärztliche Erstvorstellung der Patienten aufgrund der NF1 erfolgte bei 3 der teilnehmenden Patienten 0 bis 6 Monate vor der ersten Operation in unserem Hause (10 %). Zwei NF1-Patienten stellten sich 6 Monate bis 1 Jahr vor der Operation ärztlich vor (6,7 %). Jeweils 1 Person suchte 1 bis 2 Jahre bzw. 2 bis 5 Jahre präoperativ erstmalig einen Arzt auf (je 3,3 %). Bei der Mehrheit von 23 Personen waren es hingegen mehr als 5 Jahre (76,7 %).

Neurofibrome am Rumpf wurde bei 27 Personen (90 % der Patienten) und damit am häufigsten diagnostiziert. Auch an den Extremitäten und im Kopfbereich zeigten sich häufig Neurofibrome.

### Angepasster DLQI für die Lebensqualität prä- und postoperativ

**Figure 1:**
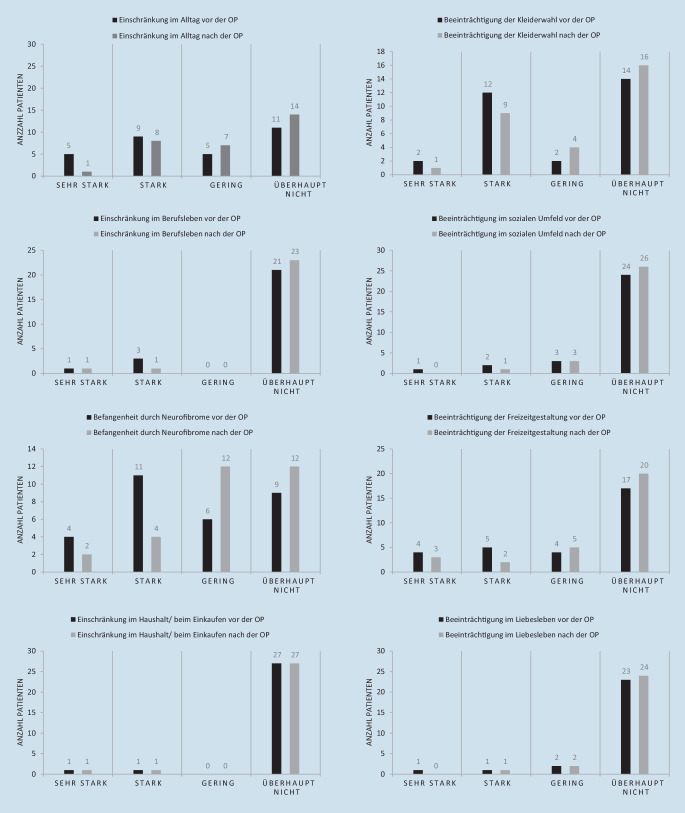

Die ausführliche numerische Auswertung der angepassten DLQIs ist Abb. 1 sowie dem Anhang zu entnehmen.

Es zeigte sich eine tendenzielle Verbesserung der allgemeinen Alltagseinschränkung durch die Operation. Zudem gaben zumindest 2 der Patienten (6,7 %) eine Verbesserung der beruflichen Einschränkung durch die Operation an, allerdings war bei 21 Patienten bereits präoperativ keine berufliche Einschränkung angegeben worden. Außerdem zeigt sich eine tendenzielle Verbesserung der Befangenheit (Gefühl der Scham, Verlegenheit durch die Neurofibrome) nach der Operation.

Einschränkungen im Haushalt wurden durch die Operation nicht verändert. Eine tendenzielle Verbesserung zeigt sich jedoch in der Beeinträchtigung der Kleiderwahl nach der Operation. Es zeigt sich eine tendenzielle Verbesserung der Beeinträchtigung der Freizeitgestaltung durch die Operation. Nach Auszählung der Rohdaten ergab sich für 4 Personen (13,3 %) eine Verbesserung im sozialen Umfeld nach der Operation angegeben, bei 3 Personen (10 %) wiederum einen positiven Effekt auf Partnerschaft und des Liebeslebens durch die Operation.

### Angepasster postoperativer Fragebogen aus unserer operativen Abteilung

Die Ergebnisse des postoperativen Fragebogens sind in Tab. 2 aufgeführt. Eine große Mehrheit von 22 Patienten (73,3 %) zeigte sich sehr zufrieden mit dem Ergebnis der Operation. Weitere 8 Personen waren zufrieden (26,7 %). Mit dem kosmetischen Ergebnis waren 27 der Patienten (90 %) sehr oder zufrieden, 3 Personen waren ausreichend zufrieden (10 %). Postoperativ gaben 12 Patienten Schmerzen im Operationsgebiet an (40 %), davon benötigten 5 Personen Schmerzmittel (16,7 % aller Probanden), wohingegen 7 Personen keine Analgetika benötigten (23,3 % aller Probanden). Die meisten befragten Patienten hatten keine weiteren Komplikationen nach der Operation (*n* = 25, 83,33 %), 4 Patienten hatten eine Nachblutung (13,3 %), 1 Patient fiel mit einem Wundinfekt auf (3,3 %).

**Table 2:** 

**–**		**Sehr zufrieden**	**Zufrieden**	**Ausreichend zufrieden**	**Nicht zufrieden**	**Sehr unzufrieden**
Zufriedenheit insgesamt mit dem Operationsergebnis		73,3 %	26,7 %	0 %	0 %	0 %
Zufriedenheit mit dem kosmetischen Operationsergebnis		46,7 %	43,3 %	10 %	0 %	0 %
**Schmerzen**			**Sonstige postoperative Komplikationen**			
*Keine*	*Schmerzmittel benötigt*	*Keine Schmerzmittel*	*Nachblutung*	*Wundinfekt*	*Funktionelle Einschränkung*	*Keine*
60 %	16,7 %	23,3 %	13,3 %	3,3 %	0 %	83,3 %
**Wiederauftreten von Neurofibromen im Operationsgebiet nach der Operation**						
*Nein*		*Ja, geringer als zuvor*	*Ja, etwa gleich*		*Ja, stärker als vor Operation*	
70 %		23,3 %	6,7 %		0 %	
**Weiterempfehlung der Operation in unserem Hause**						
*Ja*			*Nein*			
86,7 %			10 %			

Bei 70 % der Patienten (*n* = 21) wurden im Operationsgebiet postoperativ keine neu aufgetretenen Neurofibrome mehr beobachtet. Bei 10 Personen (30 %) kam es jedoch zum Wiederauftreten kutaner Neurofibrome im oder um das Operationsgebiet: 7 Patienten berichteten dabei aber von einer geringeren Anzahl an Neurofibromen verglichen zu präoperativ (23,3 %). Bei 2 Patienten war die Last an neu aufgetretenen Neurofibromen in etwa vergleichbar zum präoperativen Befall (6,7 %). Die Frage nach Weiterempfehlung einer Operation in unserem Hause beantwortete die überwiegende Mehrheit der Patienten mit ja (*n* = 26, 86,7 %).

## Diskussion

Der Einfluss der NF1 auf die Selbstwahrnehmung und Lebensqualität von Patienten ist seit Jahrzehnten in der Forschung bekannt. Zu den geläufigsten negativen Auswirkungen der Erkrankung zählen soziale Isolation sowie Probleme im Beruf. Großen Einfluss auf die Wahrnehmung der Erkrankung geht vom sozialen Umfeld aus, welches sowohl durch Stigmatisierung als auch – im positiven Sinne – durch Unterstützung auf Patienten einwirken könne [8, 9].

Insgesamt bestehe eine allgemeine Herabsetzung der Lebensqualität von NF1-Patienten gegenüber gesunden Kontrollpersonen [15].

In einer Studie von Chamseddin et al. von 2019 wurde der DLQI prä- als auch postoperativ erhoben. Der DLQI fiel dabei in allen Kategorien ab, wobei die Ergebnisse hinsichtlich Einschränkung in der Berufsausübung statistisch nicht signifikant waren [12]. Die Studie ist mit nur 12 Patienten bei insgesamt 83 operierten Läsionen von eher kleinem Umfang, dem steht allerdings der rein retrospektive Ansatz unserer Studie entgegen, welcher teilweise zu Verzerrung der Ergebnisse führen kann. Der gezeigte positive Einfluss der Exzision kutaner Neurofibrome auf die im DLQI erfragten Parameter der Lebensqualität einschließlich der Berufsausübung deckt sich aber mit unseren Ergebnissen.

Granstrom et al. untersuchten 228 NF1-Patienten mit selbstentworfenen Fragebögen. Ergebnisparameter der Studie waren Depressionen, Leidensdruck und Lebensqualität. NF1-Patienten hatten ein negativeres Körperbild; auch im Vergleich zu Patienten mit anderen entstellenden Krankheiten fühlten sich Patienten mit NF1 weniger attraktiv, generell unsicherer und waren außerdem sexuell weniger zufrieden [16]. In einer Arbeit von Kodra et al. wurden 129 Patienten je mit einem SF-36-Fragebogen und Skindex-29 befragt und deren Ergebnisse in Relation mit der Schwere der NF1 gesetzt. Hier korrelierte die Krankheitsschwere mit der subjektiven funktionellen und emotionalen Beeinträchtigung [4]. Hingegen fand sich bei Cosyns et al. keine Korrelation von Voice Handicap Index (VHI) und Dysphonia Severity Indices (DSIs) mit dem Schweregrad der NF1 [17]. Ein signifikanter Unterschied hinsichtlich des VHI bestand bei Cosyns et al. lediglich im Vergleich zur gesunden Kontrollgruppe. Männer und Frauen mit NF1 hatten dabei vergleichbare VHI-Werte, auch stieg der Gesamt-VHI-Wert mit dem Alter [17]. Das Patientenkollektiv war mit 30 NF1-Patienten aber etwas kleiner. Anders als in den Daten von Cosyns et al. ergab eine Erhebung von 128 NF1-Patienten von Wolkenstein et al., dass die befragten Frauen mit milder bis moderater NF1 einen höheren Leidensdruck (Sindex France) hatten als die befragten Männer. Allerdings liegt der Studie mit 70 % Frauen kein ausgeglichenes Geschlechterverhältnis zugrunde. Zudem wurden SF-36 und QoL (Quality of Life) erhoben, und es wurde eine schlechtere Lebensqualität der NF1-Patienten im Gegensatz zu einem Kollektiv von 3656 Gesunden aufgezeigt [18].

Zum Wiederauftreten von Neurofibromen nach Exzision existiert eine Arbeit von Needle et al., welche Fälle aus den Jahren 1974 bis 1994 umfasst. Eindrücklich ist, dass hier – je nach Resektionsstatus – ein Teil der operierten Tumoren ein Rezidiv oder weiteren Progress zeigte. Bei totaler Tumorresektion fiel dies geringer aus als bei subtotaler Resektion oder Biopsie. Auch gab es einen positiven Zusammenhang zwischen Vollständigkeit der Resektion und Latenz des Tumorprogresses oder Tumorrezidivs [10]. In unseren Daten blieben im Untersuchungszeitraum insgesamt 70 % der befragten Patienten rezidivfrei. Allerdings handelt es sich bei unserem Patientenkollektiv hauptsächlich um Erwachsene. Unser multimodaler operativer Ansatz besteht aus verschiedenen chirurgischen Techniken zur möglichst vollständigen klinisch-makroskopischen Entfernung der Neurofibrome, zum Teil auch in mehrzeitigem Vorgehen. Die Daten von Needle et al. sind insofern mit den von uns erhobenen Daten vergleichbar, dass bei (sub)totaler Tumorresektion die Mehrheit der Patienten kein erneutes Auftreten von Neurofibromen an den operierten Arealen zeigt.

Einen methodisch ähnlichen Ansatz verfolgt eine Studie von Wise et al. Hierbei handelt es sich um eine retrospektive Studie von 39 pädiatrischen Patienten, meist im präadoleszenten Alter, mit plexiformen Neurofibromen im Kopf- und Halsbereich, die aufgrund der Größe oft nicht radikal exzidiert wurden. Das krankheitsfreie Intervall nach Operation war dabei ungefähr 3 Jahre [11].

Risiken und Komplikationen der Resektion kutaner Neurofibrome fallen in unserer Studie moderat bis gering aus. Dies unterstreicht den individuellen multimodalen operativen Ansatz. Zusammen mit der hohen Zufriedenheit mit dem operativen Ergebnis, dem kosmetischen Ergebnis sowie positivem Einfluss auf verschiedene Aspekte der Lebensqualität spricht dies für ein günstiges Risiko-Nutzen-Verhältnis der operativen Resektion kutaner Neurofibrome.

### Limitationen

Unsere Studie weist Limitationen auf. So ist das Patientenkollektiv mit 30 Personen vergleichsweise klein, auch die Geschlechterverteilung ist nicht ausgeglichen und weißt zwischen den Geschlechtergruppen einen gewissen Altersunterschied auf. Eine Schweregradeinteilung der NF1 ließ sich aus der vorliegenden Patientendokumentation nicht vollständig ableiten. Zudem ergibt sich aus dem Studiendesign mit teils langen Latenzen zwischen Operation und Beantwortung des Fragebogens die Möglichkeit eines Recallbias, sodass die Patienten sich ggf. nicht mehr vollständig an ihre Operation und postoperative Komplikationen erinnern.

## Fazit für die Praxis

- Kutane Neurofibrome bei NF Typ 1 tragen v. a. an äußerlich sichtbaren oder funktionell relevanten Körperstellen zu einer Verminderung der Lebensqualität bei.
- Risiken und Komplikationen der Resektion kutaner Neurofibrome fallen in unserer Studie gering aus.
- Insgesamt stellt die operative Therapie kutaner Neurofibrome eine sichere und funktionell wie ästhetisch zufriedenstellende Therapieoption bei Patienten mit NF1 dar und sollte Patienten in Zentren angeboten werden.

### Supplementary Information




