# Supplementary material for: Cosmetic and functional results after resection of cutaneous neurofibroma in neurofibromatosis type 1: Operation of neurofibroma improves quality of life while having moderate complications
Source: Dermatologie (Heidelb). 2023 Sep 20;74(12):961–8. [Article in German] doi: 10.1007/s00105-023-05220-8 (PMC10661718; doi:10.1007/s00105-023-05220-8)
Supplement: Supplementary file 1 [file 105_2023_5220_MOESM1_ESM.pdf]

Supplementary Information: Modifizierter DLQI, Anzahl der durchgeführten Operationen mit Art des Eingriffs und Anzahl operierter Stellen insgesamt pro Patient und Modifizierter DLQI mit Darstellung der Ergebnisse prozentual anteilig an der Gesamtzahl aller Patienten

Anhang 1.1 Modifizierter DLQI

Haben Sie sich vor der Operation in unserer Klinik durch die Neurofibromatose Typ I in Ihrem Alltag eingeschränkt gefühlt?

- ☐ Ja
  - ☐ Sehr stark
  - ☐ Stark
  - ☐ Gering
- ☐ Nein

Fühlen Sie sich derzeit durch die Neurofibromatose Typ I in Ihrem Alltag eingeschränkt?

- ☐ Ja
  - ☐ Sehr stark
  - ☐ Stark
  - ☐ Gering
- ☐ Nein

Hatten Sie zu irgendeinem Zeitpunkt vor der Operation in unserer Klinik Probleme in Ihrer Berufsausübung aufgrund der Neurofibromatose Typ I?

- ☐ Ja
  - ☐ Sehr starke Probleme
  - ☐ Starke Probleme
  - ☐ Geringe Probleme
- ☐ Nein
- ☐ Keine Antwort möglich, da nicht berufstätig.

Haben Sie derzeit Probleme in Ihrer Berufsausübung aufgrund der Neurofibromatose Typ I?

- ☐ Ja
  - ☐ Sehr starke Probleme
  - ☐ Starke Probleme
  - ☐ Geringe Probleme
- ☐ Nein
- ☐ Keine Antwort möglich, da nicht berufstätig.

Haben Sie sich vor der Operation in unserer Klinik durch die Neurofibromatose befangen oder verlegen gefühlt?

☐ Ja

- ☐ Sehr stark
- ☐ Stark
- ☐ Gering

☐ Nein

Fühlen Sie sich derzeit durch die Neurofibromatose befangen oder verlegen?

☐ Ja

- ☐ Sehr stark
- ☐ Stark
- ☐ Gering

☐ Nein

Bestand vor der Operation in unserer Klinik eine Beeinträchtigung bei Einkaufen und Hausarbeit durch die Neurofibromatose?

- [ ] Ja
    - [ ] Sehr stark
    - [ ] Stark
    - [ ] Gering
  - [ ] Nein

Besteht derzeit eine Beeinträchtigung bei Einkaufen und Hausarbeit durch die Neurofibromatose?

- [ ] Ja
    - [ ] Sehr stark
    - [ ] Stark
    - [ ] Gering
  - [ ] Nein

Bestand vor der Operation in unserer Klinik eine Beeinträchtigung Ihrer Kleiderwahl durch die Neurofibromatose?

- [ ] Ja
    - [ ] Sehr stark
    - [ ] Stark
    - [ ] Gering
  - [ ] Nein

Besteht derzeit eine Beeinträchtigung Ihrer Kleiderwahl durch die Neurofibromatose?

- [ ] Ja
  - [ ] Sehr stark
  - [ ] Stark
  - [ ] Gering
- [ ] Nein

Bestand vor der Operation in unserer Klinik eine Beeinträchtigung Ihrer Freizeitgestaltung durch die Neurofibromatose?

- [ ] Ja
  - [ ] Sehr stark
  - [ ] Stark
  - [ ] Gering
- [ ] Nein

Besteht derzeit eine Beeinträchtigung Ihrer Freizeitgestaltung durch die Neurofibromatose?

- [ ] Ja
  - [ ] Sehr stark
  - [ ] Stark
  - [ ] Gering
- [ ] Nein

Bestand vor der Operation in unserer Klinik eine Beeinträchtigung bei Freundschaft, in Familie oder Partnerschaft durch die Neurofibromatose?

- ☐ Ja
    - ☐ Sehr stark
    - ☐ Stark
    - ☐ Gering
- ☐ Nein

Besteht derzeit eine Beeinträchtigung bei Freundschaft, in Familie oder Partnerschaft durch die Neurofibromatose?

- ☐ Ja
    - ☐ Sehr stark
    - ☐ Stark
    - ☐ Gering
- ☐ Nein

Bestand vor der Operation in unserer Klinik eine Beeinträchtigung Ihres Liebeslebens durch die Neurofibromatose?

- ☐ Ja
    - ☐ Sehr stark
    - ☐ Stark
    - ☐ Gering
- ☐ Nein

Besteht derzeit eine Beeinträchtigung Ihres Liebeslebens durch die Neurofibromatose?

☐ Ja

- ☐ Sehr stark
- ☐ Stark
- ☐ Gering

☐ Nein

1.2. Fragebogen

Wieviel Jahre vor der Operation in unserer Klinik traten erstmals *kutane Neurofibrome bei* der Neurofibromatose Typ I auf?

☐ 0 bis 6 Monate

☐ 6 Monate bis 1 Jahr

☐ 1 Jahr bis 2 Jahre

☐ 2 Jahre bis 5 Jahre

☐ mehr als 5 Jahre

Wieviel Monate vor der Operation in unserer Klinik stellten Sie sich wegen der Neurofibromatose Typ I zum ersten Mal bei einem Arzt vor?

☐ 0 bis 6 Monate

☐ 6 Monate bis 1 Jahr

☐ 1 Jahr bis 2 Jahre

☐ 2 Jahre bis 5 Jahre

☐ mehr als 5 Jahre

An welchen Körperstellen zeigten sich bei Ihnen kutane Neurofibrome der Neurofibromatose Typ I? (Mehrfachnennungen möglich)

☐ Gesicht

☐ Sonstige Lokalisation am Kopf

- ☐ Hals
- ☐ Rumpf
- ☐ Arme
- ☐ Beine

☐ Andere Körperstelle: ____

Teil II

Wie zufrieden waren Sie *insgesamt* mit der Operation?

- ☐ Sehr zufrieden
- ☐ Zufrieden
- ☐ Ausreichend zufrieden
- ☐ Unzufrieden
- ☐ Sehr unzufrieden

Wie zufrieden sind Sie mit dem *kosmetischen Ergebnis* der Operation?

☐ Sehr zufrieden

- ☐ Zufrieden
- ☐ Ausreichend zufrieden
- ☐ Unzufrieden
- ☐ Sehr unzufrieden

Hatten Sie nach der Operation Schmerzen im Operationsgebiet?

- ☐ Ja
  - ☐ Ich benötigte deshalb Schmerzmittel
  - ☐ Ich benötigte deshalb <u>keine</u> Schmerzmittel

☐ Nein

Traten nach der Operation Komplikationen auf?

- ☐ Ja
  - ☐ Nachblutung
  - ☐ Wundinfektion
  - ☐ Dauerhafte funktionelle Einschränkung z.B. durch Vernarbung
- ☐ Nein

Kam es seit der Operation zu einem Wiederauftreten der kutanen Neurofibrome bei Neurofibromatose Typ I in anatomischer Nähe zum Operationsgebiet?

- ☐ Ja
  - ☐ Die Beschwerden sind *geringer* als vor der Operation
  - ☐ Die Beschwerden sind in etwa *gleich* wie vor der Operation
  - ☐ Die Beschwerden sind *stärker* als vor der Operation
- ☐ Nein

Würden Sie persönlich die durchgeführte Operation bei Neurofibromatose Typ I weiterempfehlen?

- ☐ Ja
- ☐ Nein

Teil III

Haben Sie sich <u>vor der Operation</u> in unserer Klinik durch die Neurofibromatose Typ I in Ihrem Alltag eingeschränkt gefühlt?

- ☐ Ja
  - ☐ Sehr stark

- ☐ Stark
- ☐ Gering

☐ Nein

Fühlen Sie sich derzeit durch die Neurofibromatose Typ I in Ihrem Alltag eingeschränkt?

☐ Ja

- ☐ Sehr stark
- ☐ Stark
- ☐ Gering

☐ Nein

Hatten Sie zu irgendeinem Zeitpunkt vor der Operation in unserer Klinik Probleme in Ihrer Berufsausübung *aufgrund der* Neurofibromatose Typ I?

☐ Ja

☐ Sehr starke Probleme

- ☐ Starke Probleme
- ☐ Geringe Probleme

☐ Nein

☐ Keine Antwort möglich, da nicht berufstätig.

Haben Sie derzeit Probleme in Ihrer Berufsausübung *aufgrund der* Neurofibromatose Typ I?

☐ Ja

- ☐ Sehr starke Probleme
- ☐ Starke Probleme
- ☐ Geringe Probleme

☐ Nein

☐ Keine Antwort möglich, da nicht berufstätig.

Haben Sie sich vor der Operation in unserer Klinik durch die Neurofibromatose befangen oder verlegen gefühlt?

☐ Ja

☐ Sehr stark

☐ Stark

☐ Gering

☐ Nein

Fühlen Sie sich derzeit durch die Neurofibromatose befangen oder verlegen?

☐ Ja

☐ Sehr stark

☐ Stark

☐ Gering

☐ Nein

Bestand vor der Operation in unserer Klinik eine Beeinträchtigung bei Einkaufen und Hausarbeit durch die Neurofibromatose?

☐ Ja

☐ Sehr stark

☐ Stark

☐ Gering

☐ Nein

Besteht derzeit eine Beeinträchtigung bei Einkaufen und Hausarbeit durch die Neurofibromatose?

- [ ] Ja
    - [ ] Sehr stark
    - [ ] Stark
    - [ ] Gering
- [ ] Nein

Bestand vor der Operation in unserer Klinik eine Beeinträchtigung Ihrer Kleiderwahl durch die Neurofibromatose?

- [ ] Ja
    - [ ] Sehr stark
    - [ ] Stark
    - [ ] Gering
- [ ] Nein

Besteht derzeit eine Beeinträchtigung Ihrer Kleiderwahl durch die Neurofibromatose?

- [ ] Ja
    - [ ] Sehr stark
    - [ ] Stark
    - [ ] Gering
- [ ] Nein

Bestand <u>vor der Operation</u> in unserer Klinik eine Beeinträchtigung Ihrer Freizeitgestaltung durch die Neurofibromatose?

- ☐ Ja
    - ☐ Sehr stark
    - ☐ Stark
    - ☐ Gering
- ☐ Nein

Besteht <u>derzeit</u> eine Beeinträchtigung Ihrer Freizeitgestaltung durch die Neurofibromatose?

- ☐ Ja
    - ☐ Sehr stark
    - ☐ Stark
    - ☐ Gering
- ☐ Nein

Bestand <u>vor der Operation</u> in unserer Klinik eine Beeinträchtigung Ihrer Kleiderwahl durch die Neurofibromatose?

- ☐ Ja
    - ☐ Sehr stark
    - ☐ Stark
    - ☐ Gering
- ☐ Nein

Besteht derzeit eine Beeinträchtigung Ihrer Kleiderwahl durch die Neurofibromatose?

☐ Ja

- ☐ Sehr stark
- ☐ Stark
- ☐ Gering

☐ Nein

Bestand vor der Operation in unserer Klinik eine Beeinträchtigung bei Freundschaft, in Familie oder Partnerschaft durch die Neurofibromatose?

☐ Ja

- ☐ Sehr stark
- ☐ Stark
- ☐ Gering

☐ Nein

Besteht derzeit eine Beeinträchtigung bei Freundschaft, in Familie oder Partnerschaft durch die Neurofibromatose?

☐ Ja

- ☐ Sehr stark
- ☐ Stark
- ☐ Gering

☐ Nein

Bestand vor der Operation in unserer Klinik eine Beeinträchtigung Ihres Liebeslebens durch die Neurofibromatose?

- ☐ Ja
  - ☐ Sehr stark
  - ☐ Stark
  - ☐ Gering
- ☐ Nein

Besteht derzeit eine Beeinträchtigung Ihres Liebeslebens durch die Neurofibromatose?

- ☐ Ja
  - ☐ Sehr stark
  - ☐ Stark
  - ☐ Gering
- ☐ Nein

Anhang 2. Anzahl der durchgeführten Operationen mit Art des Eingriffs und Anzahl operierter Stellen insgesamt pro Patient

Anzahl OP-termine	Exzisionen	Biopsien (Punch)	Shave/Kurettage/Laser /Scherenschlag	Stellen gesamt
2	8	8	0	16
1	3	0	0	3
3	3	0	2	5
1	9	0	0	9
1	1	0	0	1
1	4	0	0	4
1	5	0	0	5
3	6	0	1	7
1	0	4	0	4
17	67	82	40	189
5	7	Anzahl n. bekannt	225+	262+
2	4	0	7	11
3	3	0	0	3
1	1	0	0	1
1	1	0	0	1
5	22	0	1	23
3	1	21	25	47
1	1	0	0	1
11	3	91	91	183
11	5	0	190	195
4	9	2	10	21
20	10	127	164	470
1	2	0	0	2
4	4	0	100	104
7	20	4	5	26
2	2	0	4	6
3	2	0	62	164
1	2	0	1	3
4	5	0	0	5
5	13	0	Anzahl n. bekannt	14+
**Gesamt**				
125	223	339+	703+	1509+

Anhang 3. Modifizierter DLQI mit Darstellung der Ergebnisse prozentual anteilig an der Gesamtzahl aller Patienten

	Sehr stark	Stark	Gering	Überhaupt nicht
Einschränkung im Alltag vor der OP	16,67 %	30 %	16,67 %	36,67 %
Einschränkung im Alltag nach der OP	3,33 %	26,67 %	23,33 %	46,67 %
	Sehr stark	Stark	gering	Überhaupt nicht
Einschränkung im Berufsleben vor der OP	3,33 %	10 %	0 %	70 %
Einschränkung im Berufsleben nach der OP	3,33 %	3,33 %	0 %	76,67 %
	Sehr stark	Stark	gering	Überhaupt nicht
Befangenheit durch Neurofibrome vor der OP	13,33 %	36,67 %	20 %	30 %
Befangenheit durch Neurofibrome nach der OP	6,67 %	13,33 %	40 %	40 %
	Sehr stark	Stark	Gering	Überhaupt nicht
Einschränkung im Haushalt/ beim Einkaufen vor der OP	3,33 %	3,33 %	0%	90%
Einschränkung im Haushalt/ beim Einkaufen nach der OP	3,33 %	3,33 %	0%	90%
	Sehr stark	Stark	Gering	Überhaupt nicht
Beeinträchtigung der Kleiderwahl vor der OP	6,67 %	40 %	6,67 %	46,67 %
Beeinträchtigung der Kleiderwahl nach der OP	3,33 %	30 %	13,33 %	53,33 %

	Sehr stark	stark	Gering	Überhaupt nicht
Beeinträchtigung der Freizeitgestaltung vor der OP	13,33 %	16,67 %	13,33 %	56,67 %
Beeinträchtigung der Freizeitgestaltung nach der OP	10 %	6,67 %	16,67 %	66,67 %
	Sehr stark	Stark	Gering	Überhaupt nicht
Beeinträchtigung im sozialen Umfeld vor der OP	3,33 %	6,67 %	10 %	80 %
Beeinträchtigung im sozialen Umfeld nach der OP	0 %	3,33 %	10 %	86,67 %
	Sehr stark	Stark	Gering	Überhaupt nicht
Beeinträchtigung im Liebesleben vor der OP	3,33 %	3,33%	6,67%	76,67%
Beeinträchtigung im Liebesleben nach der OP	0 %	3,33 %	6,67%	80%
